# Supplementary material for: Content-rich biological network constructed by mining PubMed abstracts
Source: BMC Bioinformatics. 2004 Oct 8;5:147. doi: 10.1186/1471-2105-5-147 (PMC528731; doi:10.1186/1471-2105-5-147)
Supplement: Additional File 2 — The original results of the above study (non-essential files are deleted to keep the file size under the limit set by BMC bioinformatics). [file 1471-2105-5-147-S2.bz2 › chilibotAdditionalFile2/dip05/474ID10734104E10559/html/CCL13_CCRL1.html]

 


 **CCL13** and **CCRL1** 
  
Found 14 abstracts in PubMed, retrieved 05.  
 

 What does Google say? 
 PDF only 
| .edu only 

---

**Interactive relationship** (e.g. stimulation, inhibition, etc)

**Non-interactive relationship** (e.g. studied together, co-existance, homology, etc.)

- Nanomolar concentrations of UCB35625 were sufficient to inhibit eosinophil shape change responses to MIP 1alpha, MCP 4  [ **CCL13** ] , and eotaxin, while greater concentrations could inhibit the chemokine induced internalization of both CCR1  [ **CCRL1** ]  and CCR3.  Ref: 10854442 J Biol Chem, 2000
